# The influence mechanism of relative deprivation on prosocial behavior of migrant children: based on the mediating effect of self-esteem and the moderating effect of social support

**DOI:** 10.3389/fpsyg.2025.1610985

**Published:** 2025-07-16

**Authors:** Xueqi Zhang, Fengjuan Yan, Lin Meng

**Affiliations:** ^1^School of Labor Relations, Shandong Management University, Jinan, China; ^2^School of Public Administration, Shandong Normal University, Jinan, China

**Keywords:** migrant children, relative deprivation, prosocial behavior, mediating effect, moderating effect

## Abstract

Prosocial behavior refers to a series of behaviors that are beneficial to others and social harmony, such as humility, assistance, cooperation and sharing, which are important aspects of adolescent social ability development. With the acceleration of urbanization in China, the scale of the floating population continues to expand. The structural change of “family mobility” makes migrant children become a huge group in urban society that cannot be ignored. Promoting the social integration and urban inclusion of migrant children has become a critical interdisciplinary research focus. This study examines the impact of relative deprivation on prosocial behavior among migrant children, specifically investigating the mediating role of self-esteem in this relationship and the moderating role of social support between self-esteem and prosocial behavior. Using multi-stage clustered PPS (Probability Proportional to Size) sampling, data were collected from 1,428 migrant adolescents across 12 schools in central Jinan, Shandong Province. Prosocial behavior, relative deprivation, self-esteem, and perceived social support were assessed via standardized scales. The results indicate a significant negative effect of relative deprivation on prosocial behavior among migrant children. This inhibition is partially mediated by self-esteem, while social support positively moderates the relationship between self-esteem and prosocial behavior. The findings suggest that enhancing prosocial behavior among migrant children requires proactive emotional interventions. These interventions should aim to boost self-perception and self-identity, elevate self-esteem, reduce feelings of relative deprivation, and address educational challenges. Social networks-supported multi-pronged approaches are needed to foster social integration.

## 1 Introduction

Prosocial behavior encompasses socially desirable, beneficial actions directed toward others, groups, or society. These actions include helping others, sharing resources, cooperative actions, and offering comfort or support. This behavior aims to foster positive interpersonal relationships and enhance social cohesion, ultimately contributing to personal development and social harmony (Pang et al., 2022). From an individual’s perspective, prosocial behavior serves to enhance self-esteem and realizing individual self-satisfaction. From the perspective of interpersonal relationship, prosocial behavior helps to improve interpersonal communication, promote survival adaptation and interpersonal harmony; from the perspective of whole society, prosocial behavior is a symbol of social welfare and social responsibility, and is the foundation for the harmonious development and construction of social. Since the 1870s, the development and cultivation of individual prosocial behavior has received widespread attention (Laguna et al., 2022). Research in evolutionary and developmental psychology suggests that humans’ attention to those in need and propensity for cross-group cooperation may emerge early in life. Evidence suggests that children exhibit preferences for those needing help (Geraci and Franchin, 2021), in specific contexts, tend to attribute positive, fair behaviors to individuals from racial outgroups (Geraci et al., 2024). These findings support the view that prosocial behaviors have biological and evolutionary roots, emerging from the cooperation vital for survival and human sensitivity to need and deprivation. When individuals perceive themselves as meaningful members of a group, prosocial behavior is promoted. But when social exclusion is felt, the need for positive and friendly social connection is disrupted, thereby reducing prosocial behavior (Barry and Wentzel, 2006). With the rise and development of health psychology, research on prosocial behavior has been an surged.

The urban–rural migration of rural workforce is a universal law in the industrialization and urbanization process in the world. The floating population issue has been concerned by the fields of economics, demography, sociology and management. Compared with individual mobility, the proportion of families with children is increasing, and more and more migrant workers choose to migrate with their families (Fan et al., 2011). According to survey date by the Development Research Center of the State Council, the number of migrant workers who migrated with their families accounted for 25% of the total number of migrant workers in 2010. Children of migrant workers in cities are a special group arising in the context of China’s rural–urban population migration. According to the official definition, this group refers to the school-age children and adolescents whose household registration is in the villages of other provinces (districts, cities) and counties (districts) of the province, who go to the urban areas and towns (live together) of the destination with their migrant parents and receive compulsory education at school. As of 2022, there are 13.6468 million children of migrant workers in cities enrolled in compulsory education in China, 9.6986 million in primary school and 3.9483 million in junior high school (Fan and Li, 2020). In Beijing, Shanghai, Guangdong and other concentrated areas of population inflow, the proportion of migrant children in the total number of local children even reached about 30% (Ding et al., 2020), which has become a large group in urban society that cannot be ignored.

Migrant children relocating with parents from rural to urban areas face systemic disadvantages compared to urban peers, owing to cultural disparities, contrasting lifestyles and values, and household registration restrictions. These children encounter vulnerabilities in family environments, social networks, and educational development (Zhang et al., 2019), with particular difficulties in academic integration and achievement within urban schools where discrimination and assimilation challenges are prevalent (Yang et al., 2023). A large number of literatures have studied the impact of migration on children’s growth, development and social behavior. It has been found that migrant children are more prone to feelings of inferiority, discrimination, anxiety, conflict and resistance (Chan and Ren, 2018; Herry et al., 2023), which will increase the possibility of conflict and competition in society to a certain extent (Zhang et al., 2014; Zhang et al., 2019; Ding et al., 2020), and the multiple risks faced by this group will have an impact on their prosocial behavior (Xiong et al., 2021).

After entering the adolescent stage, the migrant children begin to engage more closely with social groups, thereby acquiring skills for social adaptation (Chan and Ren, 2018). Prosocial behavior researchers have also begun to pay attention to the adolescent population. Existing research mostly focus on the developmental characteristics (Donohue and Tully, 2019), performance analysis (Beeler-Duden et al., 2022) and influencing factors (Condon, 2019)of adolescent prosocial behavior, confirming that there is a strong correlation between social comparison and prosocial behavior, particularly, “downward comparison” promotes prosocial behavior, while “upward comparison” inhibits it (Ye et al., 2021). The influencing factors are important sources for studying the mechanism of prosocial behavior. Study has found that conclusions can be roughly divided into three levels: first, family relationship such as parent–child relationship and parent–child attachment; second, individual psychological characteristics such as basic psychological needs satisfaction, sympathy and empathy; third, external environments such as peer relationship and school atmosphere. It covers subjective and objective, economic, social, emotional, as well as family, peers, and other dimensions.

Given that humans exhibit prosocial tendencies early in life, the relatively disadvantaged status of migrant children makes them highly susceptible to relative deprivation. This refers to the subjective experience where individuals or groups perceive themselves as disadvantaged through comparison with a reference group, leading to negative emotions like anger and resentment. Such negative subjective experiences, stemming from social comparison and exclusion, may constitute a key environmental stressor suppressing their inherent prosocial tendencies.

The stress-buffering model posits that both internal resources and external support are crucial factors influencing positive individual development and social adaptation (Ma et al., 2018). In recent years, self-esteem, as a mechanism of self-regulation for maintaining health and adaptation, has garnered significant research attention across disciplines. Self-esteem, defined as an individual’s overall evaluation of their own worth, is a vital protective factor for psychological resilience. High self-esteem enhances an individual’s capacity to resist stress and form a positive self-identity. The intense negative emotions accompanying relative deprivation can undermine an individual’s sense of self-worth. Consequently, higher levels of self-esteem may enable migrant children experiencing perceived relative deprivation to maintain a more positive self-cognition and sense of efficacy, buffering the impact of negative emotions. This may, to some extent, protect their prosocial behavior from excessive suppression.

Social support represents another critical protective factor urgently required by migrant children. For this population, support from society, family, and school is essential. Positive family relationships and environments, alongside constructive feedback within the home, are linked to adolescents demonstrating resilient behaviors. Similarly, supportive teacher-student and peer relationships enhance children’s psychological resilience. Research indicates that social support functions by fostering emotional security, a sense of belonging, and heightened self-esteem and confidence, while concurrently reducing anxiety and promoting physical and mental health as well as social adaptation. Consequently, social support potentially mediates the influence of self-esteem on prosocial behavior. Particularly under conditions of high social support, the protective effect of self-esteem on prosocial behavior may be amplified.

According to the Cognition-Affect-Conation Pattern, cognitive and emotional experience are the decisive factors for the generation of behavioral intention (Zeng et al., 2023). Individuals are able to generate alternative emotional responses to others and awareness of others’ feelings are the basis for the generation of behaviors directed at others (Qaisar et al., 2024). The existing researches have laid solid foundation in the analysis of “cognitive-emotion” path, but the research of “emotion-behavior” pathway is still relatively insufficient. For migrant children, a typical and large social vulnerable group, both subjective and objective factors play an important role. In addition to the objective role, the influence of their subjective emotions on behavior remains to be explored. As a micro reflection of national, social and personal qualities, as well as the internal requirements of a harmonious society, the study starts from the small starting point of migrant children to explore the influence mechanism of relative deprivation on prosocial behavior, and then triggers thinking and research on the behavior of floating population and urban integration, which has certain theoretical and practical significance.

## 2 Theoretical analysis and research hypothesis

### 2.1 The direct effect of relative deprivation on the prosocial behavior of migrant children

Relative deprivation refers to a subjective cognitive and emotional state of disadvantage or unfair inequality compared to a reference group, leading to negative emotions such as anger and dissatisfaction. This phenomenon fundamentally reflects dissatisfaction with societal conditions (Pak, 2023). When transformed into behavioral motivation, this dissatisfaction can lead to detrimental adaptations, including rejection of the prevailing social order, potentially resulting in actions that threaten public safety and disrupt social stability—behaviors distinctly opposing prosocial conduct. Studies indicate that higher individual relative deprivation correlates with lower prosocial tendencies (Callan et al., 2008). Individuals perceiving their social status as disadvantaged and their needs unmet exhibit reduced willingness to assist others. Research further suggests that individuals from higher social strata generally demonstrate more stable and elevated levels of prosocial behavior (Kraus and Callaghan, 2016). Greater access to social resources and enhanced self-control capacity among higher socioeconomic groups facilitate increased prosocial manifestations compared to lower-status individuals (Whillans et al., 2017). Vulnerable populations, such as migrant children, experience prolonged disadvantage. Social comparisons heighten their perception of unfairness, thereby intensifying relative deprivation. Relative deprivation is inversely correlated with prosocial behavior, diminishing its occurrence (Zhang et al., 2016). This deprivation also fosters interpersonal distrust, further suppressing prosocial behavior and exerting a negative influence on its expression.

The essence of relative deprivation is to reflect individuals’ dissatisfaction with society. When this emotion is transformed into a driving force of behavior, it will produce unhealthy social adaptation methods such as aversion to the social status quo, and even lead to unsafe social coping behaviors such as harming social security and disrupting social order, which is completely opposite to prosocial behaviors.

In the process of urban integration, migrant workers are involved in job-hunting, living, social assimilation, cultural adaptation and social absorption. Therefore, the influence of migrant children’s relative deprivation on their prosocial behavior can be analyzed from three dimensions: economic deprivation, social deprivation and emotional deprivation. Firstly, economic deprivation is a fundamental dimension (Tunney, 2022), which is mainly reflected in three aspects: family economic status, housing conditions and living environment. As the family members of the migrant children are mostly engaged in six industries: manufacturing, transportation, warehousing and postal industries, construction, accommodation and catering, wholesale and retail, and residential service repair (Narmaditya et al., 2023), the actual wage income is relatively lower than the average wage in the city, and the economic level is relatively poor. However, housing conditions and living costs are the dominant factors in the decision of children moving with them (Xie et al., 2024). The population at the bottom of the income class and living environment in the relocation area will migrate to obtain a stable career and higher income (Xia et al., 2024), but they will fall back to a lower level compared with the urban permanent population after moving with them. Secondly, in terms of the dimension of social deprivation, compared with urban residents, migrant workers have less social capital to access services and improve quality, and the heterogeneity is insufficient (Zheng et al., 2024b), which leads to unequal social opportunities. Such differences can easily trigger group hostility and bad interpersonal relationships. Generate behavioral coping styles that are unhealthy (such as drinking, smoking) or unsafe (such as not wearing seat belts, not exercising; Pan et al., 2024; Zheng, 2024). Finally, with the rapid development of urbanization, emotional deprivation has become an increasingly important dimension. Issues such as “exclusionary psychology,” differentiated service provision and “stigmatization” of migrant workers have become more and more prominent in cities. The social integration of migrant workers is largely constrained by these discriminatory and unfair emotions (Shen et al., 2024). The distinction between the first and second generation of migrant workers is also defined by their “emotional structure” and lifestyle (Li et al., 2024). Especially for the new generation of migrant workers, they pay more attention to emotional identity and have stronger emotional needs (Li et al., 2024).

According to existing studies, there is a significant positive correlation between relative deprivation and group bias (Palombi et al., 2024), and group bias will increase the possibility of individual or group conflicts and competition in society to some extent (Gabriel and O'Connor, 2024). Because the sense of relative deprivation of the socially disadvantaged groups is particularly strong, on the one hand, the sense of relative deprivation often leads to anger, frustration and other emotions of the individuals of the vulnerable groups, as well as low commitment to social norms (Zheng et al., 2024a), which limits the probability of the children’s prosocial behaviors such as helping, donating and volunteering. On the other hand, many migrant children consider that they belong to vulnerable groups or groups in need of help, which directly leads to their lack of intrinsic motivation for their prosocial behaviors. Furthermore, studies directly targeting migrant children have found that relative deprivation has a significant positive predictive effect on aggressive behaviors (Liu et al., 2024). Based on the above analysis, the following hypotheses are proposed:

*H*1: Relative deprivation has a negative effect on prosocial behavior.

*H*1a: Economic deprivation has a negative effect on prosocial behavior.

*H*1b: Social deprivation has a negative effect on prosocial behavior.

*H*1c: Emotional deprivation has a negative effect on prosocial behavior.

### 2.2 The mediating effect of self-esteem on the influence of relative deprivation on the prosocial behavior of migrant children

Relative deprivation arises from social comparison, typically with similar peers (Zhao and Peng, 2021). Self-esteem, a key personality factor influencing adolescent behavior, reflects an individual’s subjective evaluation of self-worth, with its level influenced by both internal and external factors. Empirical evidence indicates that relative deprivation experienced by migrant children during peer comparisons can lead to negative self-evaluations and perceptions of their environment, manifesting as diminished self-assessment and identity disturbance. This often triggers negative emotions like anger and depression. Consequently, individuals may develop negative self-judgments, ultimately damaging self-esteem (Andreassen et al., 2017). This heightened vulnerability fosters concerns about potential rejection during prosocial acts, even when well-intentioned. Such perceived or actual rejection and misunderstanding further diminish motivation for subsequent prosocial behavior.

Self-esteem can significantly relieve anxiety, depression and other negative emotional reactions (Dai and Chu, 2018), thereby inhibiting the derivative behaviors of negative emotions and promoting the generation of prosocial behaviors (Zhang et al., 2022). The analysis of its mechanism can be divided into two dimensions: self-perception and self-acceptance. On the one hand, Rosenberg proposed that self-esteem is an individual’s positive or negative attitude toward themselves, which reflects the difference between the individual’s perceived reality self-state and the ideal or expected self-state (Gong et al., 2023). He emphasized that self-esteem is an individual’s evaluation of self-worth. When an individual’s subjective perception ability and degree of value are higher, they show a more positive attitude, self-value identification is the central goal of adolescents socialization (Van Zalk et al., 2011). Migrant children with high self-esteem have higher recognition and satisfaction of their own value, lower anxiety level (Wang et al., 2022), more optimistic psychology and more positive behavior. On the other hand, Solomon believes that self-esteem is a psychological mechanism for individuals to adapt to the social and cultural environment, regulate the relationship between individuals and the environment, and affect the enthusiasm and initiative in interpersonal communication (Xiong and Hu, 2022), focusing on the definition and analysis from the dimension of self-acceptance. In the context of integration into the city, the self-acceptance of migrant children promotes the establishment of individual self-identity. It helps them to improve their courage and ability to overcome difficulties and setbacks, and enhances their sense of security and efficacy (Zhou and Zhong, 2022). Based on the above analysis, the following hypotheses are proposed:

*H*2: Relative deprivation has a negative effect on self-esteem.

*H*2a: Economic deprivation has a negative effect on self-esteem.

*H*2b: Social deprivation has a negative effect on self-esteem.

*H*2c: Emotional deprivation has a negative effect on self-esteem.

*H*3: Self-esteem has a positive effect on prosocial behavior.

Prosocial behavior is a motivational process formed by related factors such as personality, context and special conditions. The relatively unfavorable situation of migrant children has a certain negative impact on their psychology and behavior, resulting in a sense of relative deprivation, which leads to a low level of self-esteem. While self-esteem has a significant predictive effect on cognition (Fang, 2016), emotion and behavior, especially can effectively predict their prosocial behavior. The prosocial behavior of the individual is correspondingly deficient. According to existing studies, individual self-evaluation, especially self-esteem levels, can affect prosocial behavior (Moscardino et al., 2020). High self-esteem can not only help reduce the extreme tendency of individuals, but may also reduce the impact of external negative environmental factors on their behavior (Baumeister et al., 2003). Thus, when individuals develop higher self-esteem due to certain experiences, especially positive ones, have a higher level of self-esteem, their perception of the needs of other individuals will increase significantly, and the for helping others will become active and more likely to be translated into action. In this process, for migrant children with a sense of relative deprivation, the cultivation of self-esteem can enhance their relative satisfaction with themselves, thereby strengthening their self-worth perception and self-acceptance degree, reducing the possibility of participating in the cluster behavior initiated by the relative deprived individuals to change their disadvantageous position (Xiong and Hu, 2022), and then engaging in prosocial behavior. Based on the above, the following hypotheses are proposed.

*H*4: Self-esteem plays a positive mediating effect between relative deprivation and prosocial behavior.

*H*4a: Self-esteem plays a positive mediating effect between economic deprivation and prosocial behavior.

*H*4b: Self-esteem plays a positive mediating effect between social deprivation and prosocial behavior.

*H*4c: Self-esteem plays a positive mediating effect between emotional deprivation and prosocial behavior.

### 2.3 The moderating effect of social support on the influence of self-esteem on the prosocial behavior of migrant children

According to existing research, self-esteem positively promotes the generation of prosocial behaviors in individuals, and there is a positive correlation between the social support that individuals can comprehend (perceive) and their life satisfaction, subjective well-being and other positive emotions, and there is also a positive correlation between self-esteem and perceived social support (Liu et al., 2021).

According to the group characteristics of migrant children, the social support of migrant children can be divided into three dimensions: family support, peer support and other support (mainly referring to teachers, classmates and relatives). One is family support. Family is the first place that children come into contact with in the process of socialization and the main environment for their growth, which has a sustained effect on the cultivation of social skills and lifelong development (Corwin et al., 2020). The higher the family’s economic income, educational level, parent–child relationship and others, the more social resources they have and the richer social support they can obtain. The second is peer support. The support of friends and peers is the key force to help them adapt to the external environment in addition to the family. With the increasing of the age of the migrant children, the power of peer support will also increase (Wasilewski et al., 2016). However, after the migrant children come to the city, they are far away from their previous friends, facing a new living environment, their ability to cultivate friendships again needs to be improved, but the level of demand for friend support only increases and does not decrease; the third is other support. As they are in the stage of compulsory education, schools and communities are the main living and learning places, and teachers, classmates, relatives and neighbors, etc. are the groups they frequently contact on a daily basis, which play an important role in the social adaptation and integration of migrant children.

Social support is an important coping resource for migrant children to relieve stress and mitigate the negative factors brought by adversity. Its action path includes subjective and objective aspects: subjectively, it can help individuals improve their self-evaluation level, reduce the cognitive evaluation of the severity of stress and adversity, and thus reduce the existence of individual negative emotions (Lapan and Boseovski, 2017); objectively, it can provide individuals with strategies and methods to deal with problems, reducing the difficulty coefficient of stress problems (Wegmann, 2017). It can be seen that social support can enhance individual self-esteem through both subjective and objective dimensions, and at the same time, enhance the role of high self-esteem on the promotion mechanism of individual prosocial behavior. Therefore, the following hypothesis are proposed:

*H*5: Social support has a positive moderating effect between self-esteem and prosocial behavior.

**Figure 1:**
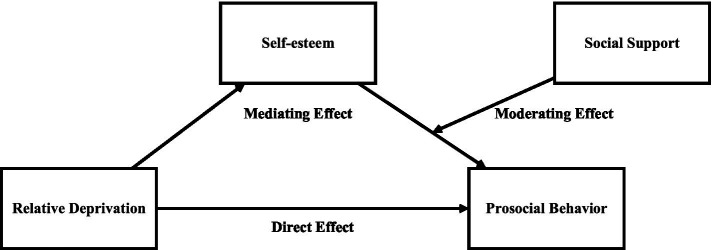
Mechanism diagram of relative deprivation effects on prosocial behavior.

Based on the above analysis, this paper attempts to integrate relative deprivation, self-esteem, social support and prosocial behavior into the same analysis framework (Figure 1), in order to provide a new perspective for the study of prosocial behavior of migrant children in the context of population mobility.

## 3 Research methods

Based on the quantitative research paradigm, this study uses questionnaire survey to collect data and information. After obtaining first-hand data, statistical software is used for empirical analysis.

### 3.1 Respondents

In this study, “Migrant children” refers to minors with rural household registration who relocate to urban areas with one or both migrant worker parents. These children must have resided in the city for over 6 months and reached compulsory education age. This defined group differs from the broader category of “mobile children,” who may originate from either rural or other urban areas. Mobile children potentially hold urban household registration themselves, and their parents may occupy high-income occupations. Consequently, migrant children constitute a specific subset of mobile children. Furthermore, “migrant children” is distinct from “children of rural migrant workers.” The latter term encompasses not only migrant children residing in cities but also left-behind children remaining in rural areas. Thus, migrant children represent only one component of the children of rural migrant workers.

Since the reform and opening up, population migration as a social phenomenon has gradually become a social normality. From a nationwide perspective, the direction of population mobility mainly shows the characteristics of flowing from rural to urban, from the west to the east. Shandong Province is the only province in China with more than 100 million permanent residents and registered population. It is a coastal province located in East China. It is a province with a large population, economy and education. Like the nationwide country, there is a regional economic development gap between the developed eastern region and the relatively backward western region. As the capital city of Shandong Province, there are about 2.103 million migrant workers in Jinan, accounting for about 40% of the permanent population, and the population inflow rate ranks among the top three in the province. Firstly, this study adopted a stratified sampling method within the city of Jinan. In this study. Before data collection, the researcher communicated with the responsible personnel of the school in advance to determine the survey time and number, and invited the migrant children of the school to participate in the questionnaire survey.

Then, according to the difference of school development level and geographical location, the migrant children of 12 schools in the central city (Lixia District, Shizhong District, Tianqiao District, Huaiyin District) were selected as the research objects. Secondly, cluster sampling method was used to conduct random sampling and comprehensive testing on the migrant children involved in the 12 schools. To minimize social desirability bias and ensure data objectivity, this study used paper questionnaires. In the process of giving out questionnaires, the researchers were either present in person or entrusted to others to provide guidance to the migrant children in filling out the questionnaire, and informed of the anonymity and confidentiality of the questionnaire information. The migrant children completed the questionnaire on site and returned it to the researchers. According to the standard of 150 questionnaires for each school, 1800 questionnaires were distributed and 1719 questionnaires were collected, of which 1,428 were valid, with an effective rate of 83.1%.

Among the 1,428 samples of migrant children, 657 were males (46%) and 771 were females (54%). Over 60% are from multi-child families. In terms of household registration types, there were 500 urban households (35%) in the province, 372 rural households (26.1%), 343 urban households in other provinces (24%), and 213 rural households (14.9%). More than 60% of the children have lived in the city with their parents for 3 years or more and were born locally. Basically, there are no frequent moves or school transfers.

In the sample of migrant children surveyed, the majority of parents have high school education (23%) and college education (29.1%). In terms of job types, manufacturing accounted for the largest proportion, followed by construction, accommodation and catering, wholesale and retail. More than half of the children believed that the overall economic status of the family was in the middle level (58.1%), which was basically consistent with the situation of China’s floating population.

### 3.2 Research tools

#### 3.2.1 Prosocial behavior questionnaire

Prosocial behavior refers to all behaviors and tendencies that individuals voluntarily make in social interactions that are beneficial to others and society (Pfattheicher et al., 2022). It is self-sacrifice made for the benefit of others or social harmony, which is manifested in humility, help, cooperation and sharing, etc. (Arbel et al., 2022), and is an important basis for maintaining a good relationship between people. It plays an important role in the healthy development of individual socialization and social adaptation. In this study, the Prosocial Tendency Scale (PTM) compiled by Carlo was used, which divided into three variables: family and social background variables, cognitive and emotional variables, and direct situational characteristics. In this study, the Prosocial Tendency Scale (PTM) compiled by Carlo was used, which divided into three variables: family and social background variables, cognitive and emotional variables, and direct situational characteristics. Based on the revised scale developed by Arbesman S and others (Peetz and Howard, 2022), design measurement indicators for prosocial behavior of migrant children from six dimensions: open, anonymous, altruistic, compliant, emotional, and urgent. The five-point Likert score method was adopted, with 1 representing “completely disagree” and 5 representing “completely agree.” The higher the score, the stronger the willingness of prosocial behavior of the migrant children. Confirmatory factor analysis showed that the structural validity index of the questionnaire (χ^2^ = 11498.507, KMO = 0.895, GFI = 0.984, AGFI = 0.978, NFI = 0.982, IFI = 0.992, CFI = 0.992, TLI = 0.990) reached an acceptable level. The Cronbach’s *α* coefficient was 0.897, indicating that the questionnaire had good reliability.

#### 3.2.2 Relative deprivation questionnaire

Relative deprivation was first proposed by Stoffer, Suchman, De Vinney, Star and Williams in 1949 (Christ et al., 2016), and then systematically explained by sociologist Merton in his book Social Theory and Social Structure (Li and Zhang, 2023). Relative deprivation is a kind of subjective psychological feeling and state of an individual or group. By comparing with the reference group, a kind of self-interest is deprived by other groups, that is, the feeling of being in a disadvantaged position (Gaskell and Smith, 1984). With reference to Hyunji K’s research (Kim et al., 2018), this study divides relative deprivation into three dimensions: economic deprivation, social deprivation and emotional deprivation. The economic dimension mainly refers to family economic status, housing conditions and living environment, the social dimension mainly refers to the interpersonal relationship status, behavioral adaptation and other aspects, and the emotional dimension is reflected in the discrimination and unfairness felt by the migrant children. The five-point Likert scale was used, with 1 representing “completely disagree” and 5 representing “completely agree,” and the higher the score, the stronger the sense of relative deprivation felt by the migrant children. Confirmatory factor analysis showed that the structural validity index of the questionnaire (χ^2^ = 4984.283, KMO = 0.788, GFI = 0.993, AGFI = 0.987, NFI = 0.991, IFI = 0.996, CFI = 0.996, TLI = 0.994) reached the acceptable level. The Cronbach’s *α* coefficient was 0.8, indicating that the questionnaire had good reliability.

#### 3.2.3 Self-esteem questionnaire

Self-esteem is a kind of personal value judgment, expressed as an individual’s attitude toward oneself (Gong et al., 2023). Its function is to monitor the degree to which individuals are accepted and rejected by others, and to motivate people to act in a way that minimizes the possibility of exclusion or rejection (Sekscinska et al., 2021). Self-esteem is the core of personality traits and also the core of individual mental health. In this study, the Self-Esteem Scale (SES) compiled by Rosenberg (1965) was used to assess the overall perception about self-worth and self-acceptance among migrant children. However, due to differences in understanding between Chinese and Western cultures, the eighth item of the original scale, “I hope I can earn more respect for myself,” was deleted with reference to suggestion of Xiaoyu C et al. (Chen and Cheng, 2023), and the valid data of 9 items were finally obtained. The five-point Likert scale was used, with 1 representing “completely disagree” and 5 representing “completely agree,” and the higher the score, the higher the self-esteem of the migrant children. Confirmatory factor analysis showed that the structural validity index of the questionnaire (χ^2^ = 4160.759, KMO = 0.867, GFI = 0.989, AGFI = 0.980, NFI = 0.985, IFI = 0.990, CFI = 0.990, TLI = 0.985) reached an acceptable level. The Cronbach’s *α* coefficient was 0.842, indicating that the questionnaire had good reliability.

#### 3.2.4 Social support questionnaire

Social support refers to the selective mechanism by which the society helps the weak free of charge through material or spiritual means, including objective support and subjective support. It is the aggregation of family, friends and social institutions that people rely on to meet their social, physical and psychological needs. Understanding social support refers to an individual’s expectation and evaluation of actual social support (French et al., 2018). In this study, the Perceived Social Support Scale (PSSS) compiled by Zimet et al. and revised by Kocalevent R et al. (Audrin and Audrin, 2023), combined with the characteristics of migrant children and referring to the modifications made by Di L et al. (Mao et al., 2021), the original “leader, relative, colleague” in the scale was changed to “teacher, classmate, relative.” Finally, it consists of three sub-scales: family support, peer support and other support (teachers, classmates and relatives), so as to reflect the degree of support from family, friends and other aspects felt by the migrant children, and reflect the total social support felt by the individual. Using a Likert five-point scale, higher scores indicate more social support for migrant children. Confirmatory factor analysis showed that the structural validity index of the questionnaire (χ^2^ = 7917.563, KMO = 0.902, GFI = 0.980, AGFI = 0.969, NFI = 0.979, IFI = 0.985, CFI = 0.985, TLI = 0.980) reached an acceptable level. The Cronbach’s α coefficient was 0.885, indicating that the questionnaire had good reliability.

## 4 Empirical results and analysis

In this study, SPSS 22.0 and AMOS 26.0 were used to conduct empirical analysis of the data, and structural equation model was used to conduct empirical research on the impact and mechanism of relative deprivation of migrant children on their prosocial behavior. Meanwhile, Bootstrap regression path analysis was employed to test the mediating and moderating effects by incorporating all four variables into a single model.

### 4.1 Common method deviation test

Using questionnaire to collect data will inevitably lead to the problem of common method bias. In order to control the common method bias, this study adopted the program control method to reduce the common method bias from the source as much as possible, including ensuring the anonymity of the questionnaire answers, and not arranging the items in the order of variables. Hermann single factor analysis was also used in this study to test the common method bias of the collected data. The exploratory factor analysis results without rotation extracted a total of 14 factors with feature roots greater than 1, and the maximum variance explained by a single factor was 22.6% (<40%), indicating no severe common method bias.

### 4.2 Description and correlation analysis of each variable

The mean, standard deviation, maximum, minimum and correlation coefficient of the main variables in this study are shown in Table 1. When a connection between variables exists but causality cannot be directly inferred, the relationship is called correlation. Firstly, this paper analyzed the relationship between variables in this study through Person correlation. Among them, relative deprivation was negatively correlated with the four main variables of prosocial behavior, self-esteem and social support, which were consistent with the expectations of this study and provide preliminary support for subsequent hypothesis testing.

**Table 1:** Description and correlation analysis of main variables.

Items	M	SD	MIN	MAX	Economic deprivation	Social deprivation	Emotional deprivation	Self-esteem	Social support	Prosocial behavior
Economic Deprivation	2.483	0.878	1	5	1	0.325**	0.295**	−0.274**	−0.356**	−0.361**
Social Deprivation	2.540	0.912	1	5	0.325**	1	0.229**	−0.184**	−0.184**	−0.342**
Emotional Deprivation	2.773	0.966	1	5	0.295**	0.229**	1	−0.283**	−0.283**	−0.219**
Self-Esteem	3.010	0.938	1	5	−0.274**	−0.184**	−0.283**	1	0.504**	0.323***
Social Support	3.494	0.982	1	5	−0.356**	−0.293**	−0.233**	0.504**	1	0.483**
Prosocial Behavior	3.156	0.922	1	5	−0.361**	−0.342**	−0.219**	0.323**	0.483**	1

*p < 0.05, **p < 0.01, ***p < 0.001.

### 4.3 Hypothesis testing based on structural equation model

On the basis of the correlation analysis of the variables, this section will use the structural equation model to deeply explore the relationship between the four variables.

#### 4.3.1 Hypothesis testing of mediating effect

**Figure 2:**
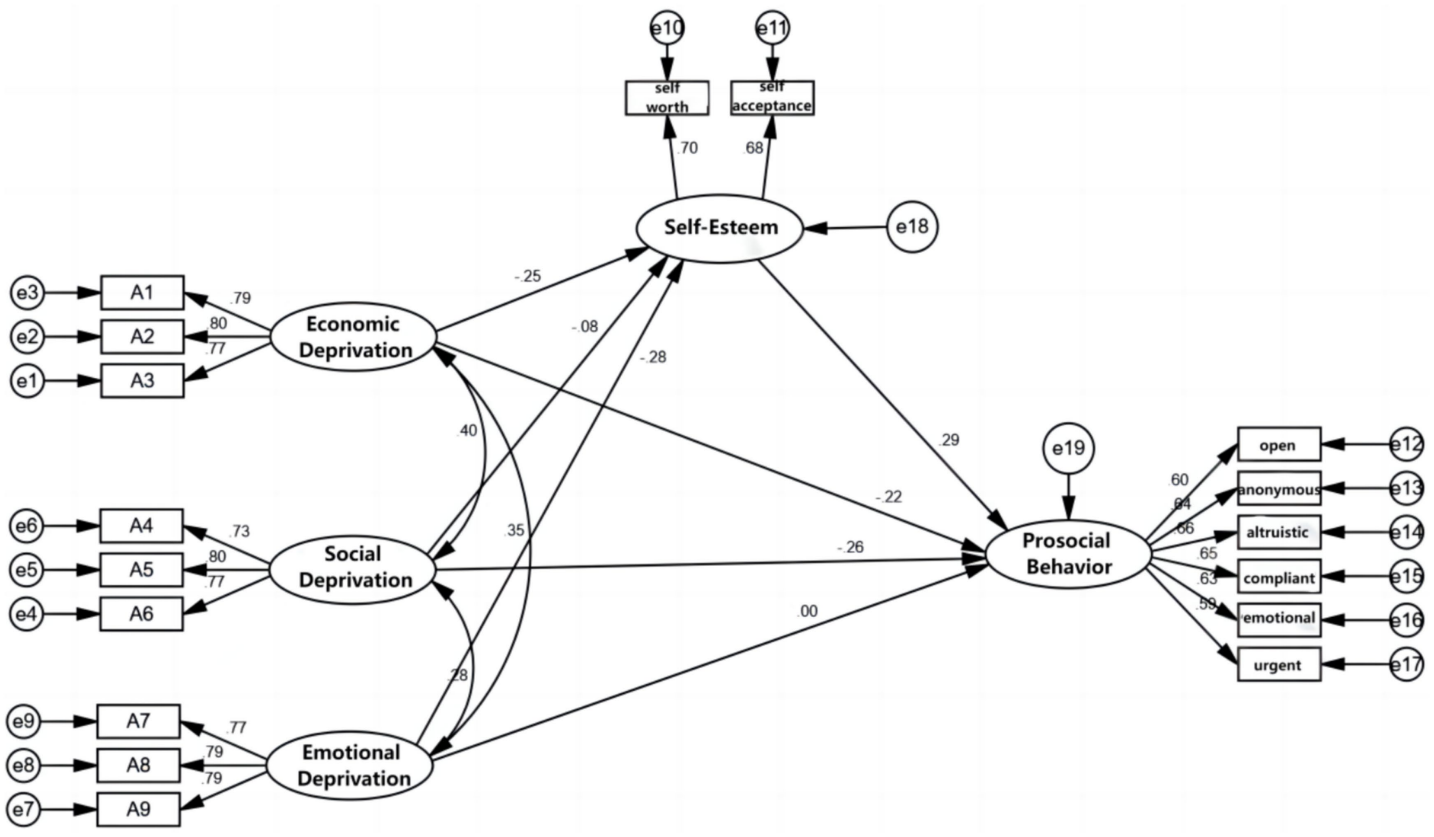
Breakdown of self-esteem mediation effect test.

In order to verify the direct effect of relative deprivation on prosocial behavior and the mediating effect of self-esteem in migrant children, this study constructed a structural equation model with relative deprivation as the independent variable, self-esteem as the mediating variable, and prosocial behavior as the dependent variable (see Figure 2). The model fitting index was as follows: χ^2^/df = 4.437, RMSEA = 0.049, NFI = 0.942, GFI = 0.961, AGFI = 0.945, NFI = 0.942, IFI = 0.955, CFI = 0.955, TLI = 0.943. The model fitting indexes were all within the recommended value range. It indicates that the structural equation model established in this study is effective and well matched with the recycled data, and can be further explained by the model.

In structural equation model, path coefficient reflects the influence relationship and degree between variables (Asparouhov et al., 2018). As shown in Figure 2 and Table 2, the three dimensions of relative deprivation had significant impact on the direct path of prosocial behavior, among which economic deprivation had an impact on the direct path of prosocial behavior (*γ* = −0.222, *p* = <0.001), indicating that economic deprivation had a significant negative impact on prosocial behavior, hypothesis H1a was valid. The direct path of social deprivation on prosocial behavior was significant (γ = −0.258, *p* = <0.001), indicating that social deprivation had a significant negative effect on prosocial behavior. Hypothesis H1b was valid. The standardized path coefficient of affective deprivation on prosocial behavior was −0.001 (*t* = −0.015, *p* = 0.988 > 0.05), indicating that emotional deprivation had no significant impact on prosocial behavior, so the hypothesis H1c was not valid.

**Table 2:** Path verification of self-esteem mediation model.

Path	γ	S. E.	C. R.	*p*
Self-Esteem←Economic Deprivation	−0.246	0.035	−5.948	***
Self-Esteem←Social Deprivation	−0.08	0.033	−2.04	0.041
Self-Esteem←Emotional Deprivation	−0.277	0.029	−7.018	***
Prosocial Behavior←Economic Deprivation	−0.222	0.026	−5.99	***
Prosocial Behavior←Social Deprivation	−0.258	0.024	−7.281	***
Prosocial Behavior←Emotional Deprivation	−0.001	0.021	−0.015	0.988
Prosocial Behavior←Self-Esteem	0.294	0.036	6.748	***

*p < 0.05, **p < 0.01, ***p < 0.001.

Relative deprivation can significantly negatively predict self-esteem, with economic deprivation having a significant direct impact on self-esteem (γ = −0.246, *p* < 0.001), indicating that economic deprivation had a significant negative impact on self-esteem, hypothesis H2a was valid; Social deprivation had a negative impact on self-esteem (γ = − 0.08, *p* < 0.05), assuming H2b was valid; The direct path of emotional deprivation on self-esteem was significant (γ = −0.277, *p* < 0.001) indicates that emotional deprivation had a significant negative impact on self-esteem, therefore hypothesis H2c was valid. And after verification, self-esteem can significantly negatively predict prosocial behavior (γ = 0.294, *p* < 0.001). Therefore, self-esteem may play a mediating role between relative deprivation and prosocial behavior.

This study used bootstrap sampling method and conducted 2000 repeated sampling tests to examine the mediating effect at a 95% confidence interval. The self-sampling method estimates indirect effects and sampling distribution through repeated sampling, and estimates the confidence interval of indirect effects based on the characteristics of data distribution, which is more scientific and accurate, and has been recognized by researchers (Kusurkar et al., 2013). The results are shown in Table 3. In the mediating path of economic deprivation dimension, the total effect value was −0.295, the upper and lower intervals of 95% confidence interval were negative, excluding 0, and the *p*-value was less than the significant level 0.05, indicating the existence of the total effect. The direct effect value was −0.222, the upper and lower intervals of 95% confidence interval were negative, excluding 0, and the *p*-value was less than the significant level 0.05, indicating the existence of direct effect, accounting for 75.2% of the total effect. The indirect effect value was −0.072, with 95% confidence interval limits both negative and excluding zero, and the *p*-value was less than the significant level 0.05, indicating the existence of indirect effect, accounting for 24.4% of the total effect. Hypothesis H4a was valid. In the mediating path of social deprivation dimension, the total effect value was −0.281, the upper and lower intervals of 95% confidence interval were negative, excluding 0, and the *p*-value was less than the significant level 0.05, indicating the existence of the total effect. The direct effect value was −0.258, the upper and lower parts of 95% confidence interval were negative, excluding 0, and the *p*-value was less than the significant level 0.05, indicating the existence of direct effect, accounting for 91.8% of the total effect. The indirect effect value was −0.024, and the upper and lower parts of 95% confidence interval were positive and negative, including 0, and the *p*-value was greater than the significant level 0.05, indicating that the indirect effect did not exist. Therefore, it is proved that hypothesis H4b was not valid. In the mediating path of emotional deprivation, the total effect value was −0.082, the upper and lower intervals of 95% confidence interval were negative, excluding 0, and the *p*-value was less than the significant level 0.05, indicating the existence of the total effect. The direct effect value was −0.001, the upper and lower parts of 95% confidence interval were positive and negative, including 0, and the *p*-value was greater than the significant level 0.05, indicating that the direct effect did not exist. The indirect effect value was −0.081, the upper and lower parts of 95% confidence interval were positive, excluding 0, and the *p*-value was less than the significant level 0.05, indicating the existence of indirect effect, accounting for 98.9% of the total effect. Hypothesis H4c was valid. Since the independent variable has no direct impact on the dependent variable, it is a complete intermediary.

**Table 3:** Bootstrap test of mediation effect.

Path	Type	Value	SE	95%CI		*p*	Ratio
				Lower	Upper		
Path 1: Economic Deprivation-Self-Esteem-Prosocial Behavior	Total Effect	−0.295	0.044	−0.379	−0.207	0.000	-
	Direct Effect	−0.222	0.047	−0.314	−0.128	0.000	75.2%
	Indirect Effect	−0.072	0.019	−0.117	−0.041	0.000	24.4%
Path 2: Social Deprivation-Self-Esteem-Prosocial Behavior	Total Effect	−0.281	0.038	−0.354	−0.207	0.000	-
	Direct Effect	−0.258	0.038	−0.33	−0.182	0.000	91.8%
	Indirect Effect	−0.024	0.014	−0.054	0.002	0.065	-
Path 3: Emotional Deprivation-Self-Esteem-Prosocial Behavior	Total Effect	−0.082	0.039	−0.16	−0.005	0.034	-
	Direct Effect	−0.001	0.038	−0.073	0.077	0.989	-
	Indirect Effect	−0.081	0.018	−0.124	−0.05	0.000	98.9%

#### 4.3.2 Hypothesis testing of moderating effect

To examine the effect of social support on self-esteem and prosocial behavior, self-esteem was taken as an independent variable, prosocial behavior as a dependent variable, and social support as a moderating variable. Since self-esteem, social support and prosocial behavior are all latent variables, the moderating effect analysis of latent variables was used to examine the moderating effect of social support (Yuan and Zhang, 2020). Firstly, each dimension of the three variables is processed centrally. Secondly, the index of the interaction term is generated. Finally, the structural equation model of latent variable interaction was constructed to test whether the moderating effect of social support was significant.

As shown in Table 4, Model 1 establishes a multiple regression model with self-esteem and perception of social support as independent variables and prosocial behavior as dependent variables. Model 2 was a multiple regression model established with self-esteem, social support perception and interaction item self-esteem * social support perception as independent variables, and prosocial behavior as dependent variables. In model 1, independent variable self-esteem had a significant negative effect on prosocial behavior (*β* = 0.107, *t* = 4.015, *p* < 0.001). The regression coefficient of the interaction term of the independent variable and the moderating variable in model 2 was 0.056 (*t* = 2.088, *p* < 0.05), indicating that the interaction term had a significant positive impact on prosocial behavior, and the R^2^of model 1 was 0.242, and that of model 2 was 0.244, which was significant improvement, indicating that the explanatory power of the model was enhanced.

**Table 4:** The moderating effect of social support perception on self-esteem and prosocial behavior.

Variable	Model 1		Model 2	
	*β*	*t*	*β*	*t*
Self-esteem	0.107	4.015***	0.121	4.395***
Social Support Perception	0.429	16.053***	0.447	15.905***
Self-esteem * Social Support Perception	-	-	0.056	2.088*
R^2^	0.242		0.244	
Adjusted R^2^	0.241		0.242	
△R^2^	0.242		0.002	
F	227.057***		153.181***	

*p < 0.05, **p < 0.01, ***p < 0.001.

**Figure 3:**
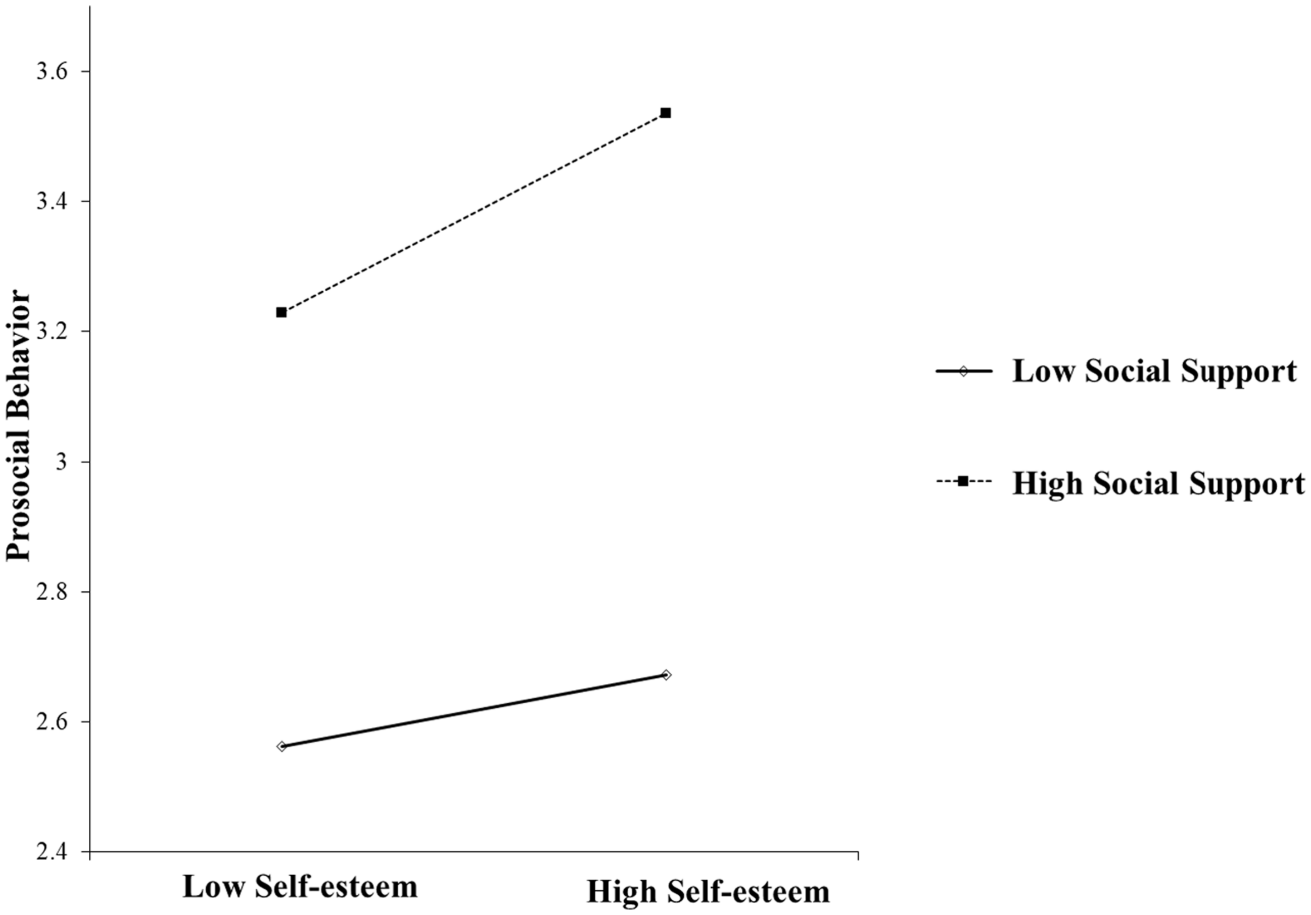
Moderating effect diagram.

To further understand the moderating effect of social support on self-esteem and prosocial behavior of migrant children, a simple slope analysis was conducted. The effect value of the high group was 0.676, the interval was (0.538, 0.876), *p* < 0.001; the effect value of low group was 0.371, and that of interval was (0.281, 0.459), *p* < 0.001. The change in the slope plot was shown in Figure 3, compared with the migrant children with low self-esteem, the predictive effect of relative deprivation on prosocial behavior is stronger in the migrant children with high social support. Therefore, it proves that the moderating variable perceived social support has a significant positive moderating effect on the relationship between self-esteem and prosocial behavior, and hypothesis H5 is valid.

## 5 Discussion and conclusion

### 5.1 Discussion

Based on the micro-survey data of 1,428 migrant children in Shandong Province, this paper uses exploratory factor analysis and Bootstrap moderated mediation test methods to empirically research the internal mechanism of the impact of relative deprivation on prosocial behavior. The study found:

Firstly, the dimensions of economic deprivation and social deprivation in relative deprivation have a significant negative effect on prosocial behavior, inhibiting the prosocial behavior of migrant children, which is basically consistent with similar research results at home and abroad (Xiong and Hu, 2022; Zou and Xiong, 2023). On one hand, economic scarcity not only restricts migrant children’s participation in financially demanding prosocial activities (e.g., donations, group outings), but more importantly, it fosters intense feelings of inequity and resource anxiety through frequent, unavoidable social comparisons regarding material circumstances and consumption levels. This persistent state of ‘relative poverty’ heightens ego depletion, promoting resource conservation over contribution, thereby weakening prosocial motivation and capacity (Woo et al., 2022). On the other hand, perceived social deprivation primarily stems from migrant children’s experience of systemic exclusion and marginalization within institutional structures, cultural acceptance, and social network resources. This sense of deprivation undermines their belonging and trust in the broader social community. When individuals perceive systematic neglect or exclusion by mainstream society, their identification with social norms and reciprocity principles diminishes. An ‘us-versus-them’ group boundary mentality develops, consequently reducing prosocial inclinations toward out-groups.

Contrary to expectations, the direct negative effect of emotional deprivation on prosocial behavior was not statistically significant in this study. This suggests a highly complex relationship between emotional experiences and behavioral responses among migrant children (Surian et al., 2025). In terms of emotional dimension, migrant children undoubtedly have more negative emotions and less positive emotions than urban children. The identity of migrant workers does increase the perception of discrimination among migrant children to a certain extent, making them more sensitive to their household registration status. However, according to existing studies, perception of discrimination involves whether a potential victim attributes the behavior of others to bias (Major et al., 2002), and the negative effect of discrimination attribution tendency on the feelings of the migrant children is relatively weak. It has been verified that factors such as personal control, belief in a just world, and self-esteem play mediating roles in the influence path of the negative feelings of the migrant children (Esposito et al., 2023). Especially when the belief in a just world is high, the negative influence of relative deprivation on the mental health level is weak. Moreover, due to the kindness, simplicity and sincerity in the “local” attributes of migrant children, the occurrence of prosocial behavior can be rooted in the nature to a certain extent (Zhu et al., 2023).

Secondly, self-esteem plays a negative mediating role in the inhibition of prosocial behavior of relative deprivation, which is mainly reflected in the dimensions of economic deprivation and emotional deprivation. For a long time, domestic and foreign studies on Chinese urban migrant children have mainly been based on the perspective of the deficit model, which believes that migrant children have more or less certain development problems in adverse situations. With the rise of positive development, people gradually realize that, migrant children also have a positive development of psychological state, namely self-esteem, so that even in adverse circumstances, the individual still has the potential or advantage of healthy development (Wang et al., 2022). Significant perceived disadvantage by migrant children relative to their reference group in economic resources, emotional support, or social status triggers intense feelings of undeservingness and devaluation. This persistent negative self-appraisal directly undermines core self-esteem. Reduced self-esteem signifies a substantial depletion of the psychological energy required to maintain a positive self-image, resist external pressures, and engage proactively in society. As prosocial behavior typically demands additional psychological resources, individuals with depleted resources due to impaired self-esteem tend to adopt more conservative, self-protective strategies, significantly suppressing both the willingness and likelihood of engaging in prosocial acts.

Furthermore, this study identified that the mediating effect of self-esteem was particularly concentrated and significant for perceived economic and emotional deprivation. Economically, for adolescents at a critical developmental stage, significant economic disparities are not only associated with material deprivation but are frequently internalized as indicators of personal or familial inadequacy and labels of being a “failure” in social comparisons” (Ding and Wu, 2023). This stigmatizing perception readily translates into fundamental self-doubt, strongly eroding self-esteem. Under low self-esteem, individuals may become more focused on fulfilling their own needs, exhibiting significantly reduced empathy toward others’ difficulties and diminished willingness to help. Emotionally, the adolescent period demands intense belongingness and emotional support. Perceived lack of emotional support within the family or peer group directly undermines core beliefs of being “loved” and “accepted,” provoking profound loneliness and self-denial. This rupture in emotional connection often delivers a swift and deep blow to self-esteem. Individuals with low self-esteem find it harder to establish and maintain positive social relationships, consequently suppressing prosocial motivation.

In contrast, the mediating effect of perceived social deprivation on self-esteem may be relatively weaker. This suggests that deprivation in the social dimension is more often attributed to structural or systemic inequities external to the individual, rather than being wholly ascribed to personal inadequacy or worth. Consequently, its direct impact on core self-esteem is likely less intense and individualized than that of economic or emotional deprivation. However, this does not imply that social deprivation is unimportant; its influence may operate through other, more complex pathways—such as learned helplessness, eroded social trust, or threats to group identity—to affect behavior, or exert long-term, cumulative negative effects on self-esteem.

Thirdly, social support plays a positive regulating role between self-esteem and prosocial behavior, that is, the more social support migrant children perceive, the stronger the effect of self-esteem on prosocial behavior, and vice versa. This finding verifies children’s needs for the external environment in the growth stage. When children have a positive affirmation of themselves, they need to be strengthened through the support and help of the external environment and then transformed into behaviors. First, migrant children, due to their mobile background, frequently encounter relative resource scarcity during adaptation to new environments, fostering feelings of relative deprivation. Adequate social support serves as a critical compensatory mechanism for psychological resources at this juncture. Emotional care, instrumental assistance, and validation from significant others directly provide individuals with additional psychological energy and security. These external resources effectively empower individuals, enabling them to overcome internal depletion potentially caused by relative deprivation or past negative experiences. Consequently, the intrinsic positive power of self-esteem is more fully and confidently manifested as prosocial behavior. Second, support at both school and family levels facilitates the positive integration of social identity among migrant children (Baysu et al., 2023). When migrant children perceive recognition and respect for their membership within the new environment, their sense of belonging and responsibility toward the group strengthens. This integrated positive social identity significantly enhances their motivation to translate intrinsic self-esteem into behaviors beneficial to the group, since prosocial conduct is then viewed as an effective means of safeguarding and enhancing collective well-being.

### 5.2 Conclusion and suggestions

This study adopts an empirical research approach, incorporating both mediating and moderating mechanisms into the same research framework, revealing the internal process of the impact of relative deprivation on the prosocial behavior of migrant children. Based on the current era background of our country, this paper puts forward relevant countermeasures and suggestions based on empirical results.

#### 5.2.1 Carry out active guidance to weaken relative deprivation

As a special vulnerable group, migrant children’s relative deprivation caused by comparison can easily lead to psychological problems and inhibit the emergence of prosocial behavior. On the one hand, it is necessary to actively guide migrant children to make reasonable social comparisons, such as avoiding blind comparison or selecting inappropriate comparison objects; On the other hand, when migrant children have experienced relative deprivation, they should pay attention to providing reasonable guidance for their negative emotions such as unfairness, dissatisfaction, frustration, and anger to avoid emotional disorders. For the urban society, especially the government of the destination, it is necessary to promote the reform of the household registration system, reduce the gap between urban and rural areas, and effectively protect the right of migrant children to receive education.

#### 5.2.2 Shape identity and enhance self-esteem

Ensuring equal access to compulsory education for children of migrant workers constitutes a crucial component of achieving educational equity. Currently, the national “two-pronged approach” policy and the revised Compulsory Education Law provide solid institutional guarantees for the legal status and educational rights of these children. This fundamentally mitigates their sense of social deprivation and establishes a legal foundation for constructing a positive identity. However, merely guaranteeing school enrollment opportunities is insufficient. Beyond implementing identity education in public schools to enhance their sense of fulfillment through educational integration and reduce self-deprecation stemming from identity differences—thus directly elevating their self-esteem—awareness should be raised regarding the need for psychological courses and counseling for children of migrant workers in private schools. Furthermore, a certain number of professional psychological counselors must be allocated to these schools. Additionally, structured social skills development activities should be conducted to assist these children in developing positive psychological attributes. This involves fostering a developmental perspective on self, establishing reasonable beliefs, learning to recognize personal strengths, and continuously reinforcing their self-esteem levels.

#### 5.2.3 Strengthen social support and promote urban integration

Whether migrant children can “enter, stay and learn well” is an important problem that restricts their urban integration. Therefore, unfair treatment and discriminatory policies toward migrant children should be avoided in the whole process, and unfair treatment and discrimination against migrant children should be eliminated as far as possible. Ensure timely and smooth school enrollment for migrant children. In terms of enrollment standards, on the one hand, reduce the enrollment conditions of migrant workers’ children, simplify the enrollment procedures of migrant workers’ children, improve the efficiency of handling, and create a social support atmosphere; In terms of peer support, strengthen the shaping of campus and class culture, create a good atmosphere of harmony, friendship and mutual help, and strengthen the guidance of family support, ultimately building a five-in-one care and support system for migrant children of “individual-family-school-society-government.”

#### 5.2.4 Enrich intervention pathways and stimulate prosocial behaviors

Adolescents are the critical period of life development and the cultivation of good social behaviors. The education practice of migrant children is influenced by many factors during their growth process, so it is very important to strengthen intervention. At the source, the possibility of relative deprivation should be reduced, the self-esteem level should be enhanced through the positive guidance of the process, and the identity recognition should be strengthened. At the same time, the family, peer and other comprehensive social support system should be built, and the prosocial behavior of migrant children should be shaped by demonstration, correction, imitation and other mechanisms. In addition, the prosocial behaviors of migrant children should also be protected and advocated, and the prosocial behaviors with wide influence and great effectiveness should be publicized and commended, so as to encourage the diffusion and promotion of prosocial behaviors.

### 5.3 Research prospects

This study adopts quantitative research method, through a certain scale questionnaire survey, to explore the impact and mechanism of relative deprivation on the prosocial behavior of migrant children. Although rigorous attempts were made to maintain objectivity during the research process, this study nevertheless has inherent limitations and shortcomings.

Limitations exist regarding the research methodology, specifically the use of cross-sectional data for hypothesis testing. While the findings align with theoretical predictions, the causal relationships between variables were not further verified using longitudinal or experimental methods. Furthermore, participants were recruited exclusively from migrant children attending 12 schools in Jinan City, Shandong Province. Consequently, the sample cannot be generalized to the broader migrant children population across different geographical regions. Future research should employ longitudinal data or experimental methods to enhance the model’s robustness. Expanding both the scope of participants and their geographic diversity is necessary to enhance external validity.

## Data Availability

The raw data supporting the conclusions of this article will be made available by the authors, without undue reservation.
